# Expression of peroxiredoxin-6 in the epididymal microenvironment and sperm of sheep

**DOI:** 10.5194/aab-67-393-2024

**Published:** 2024-08-13

**Authors:** Jiaoxia Xu, Jian Zhang, Yukun Song, Gaowa Hasi, Zhaojin Luan, Wei Du, Jiaxin Zhang

**Affiliations:** 1 College of Animal Science, Inner Mongolia Agricultural University/ Inner Mongolia Key Laboratory of Sheep & Goat Genetics, Breeding and Reproduction, Hohhot, Inner Mongolia, 010018, China

## Abstract

Sperm complete their maturation in the epididymis. Mature sperm are highly sensitive to oxidative damage. Peroxiredoxin-6 (PRDX6) is an important antioxidant enzyme. In this study, we investigated PRDX6 expression in the epididymal microenvironment and its distribution in the sperm of sheep. We found that PRDX6 mRNA and protein had the highest expression in the caput epididymis, followed by the corpus epididymis and cauda epididymis ( p<0.01 ). PRDX6 protein expression in epididymal fluid was higher in the caput epididymis than in the corpus epididymis and cauda epididymis ( p<0.01 ). Similarly, PRDX6 protein expression was higher in sperm derived from the caput epididymis and corpus epididymis than in sperm derived from the cauda epididymis ( p<0.01 ). Immunofluorescence revealed that PRDX6 was present only in the head of sperm derived from the caput epididymis and corpus epididymis but was distributed within the principal and middle regions of sperm derived from the cauda epididymis. Furthermore, PRDX6 was present in all parts of ejaculated sperm. In conclusion, PRDX6 showed a wider distribution in sperm cells during transport through the epididymis, and PRDX6 expression levels in epididymal tissue, epididymal fluid, and epididymal sperm decreased from the caput epididymis to the cauda epididymis. These results suggest that PRDX6 has an important role during sperm maturation in the epididymis.

## 1 Introduction

Sperm are incapable of movement and fertilization immediately after being produced in the testicles (Sullivan et al., 2019). The epididymis is the primary site for sperm maturation (Baskaran et al., 2019), and epididymal maturation is a key stage in the formation of healthy sperm in mammals (Baskaran et al., 2019; Bedford, 1994). The epididymal epithelium secretes proteins into the epididymal fluid that act directly or indirectly to promote sperm maturation (Gervasi and Visconti, 2017; Luan et al., 2021).

As sperm cells mature in the epididymis, their cytoplasm content decreases while the droplet gradually falls off. During this process, there is a corresponding increase in the polyunsaturated fatty acid content of the sperm plasma membrane, which makes mature sperm particularly sensitive to reactive oxygen species (ROS; O'Flaherty, 2018; Juárez-Rojas et al., 2022). In general, ROS in the epididymis are produced not only by the aerobic metabolism of sperm cells (O'Flaherty and de Souza, 2011) but also by epididymal epithelial cells. If the ROS levels exceed the normal physiological range, the sperm are subjected to oxidative stress (Morielli and O'Flaherty, 2015; Ribeiro et al., 2022), which can affect their motility (Morielli and O'Flaherty, 2015) and ability to bind to oocytes (Aitken et al., 1998). Normally, antioxidant enzymes are secreted into the epididymal fluid by epithelial cells, and the cooperative action of multiple antioxidant enzymes, including peroxiredoxins (PRDXs), glutathione peroxidases (GPXs), catalase (CAT), and superoxide dismutase (SOD), is needed to protect maturing sperm (O'Flaherty, 2019; James et al., 2020; Liu et al., 2022). How these antioxidant enzymes interact with each other remains to be investigated.

PRDXs are a family of antioxidant enzymes that can be divided into two subtypes: 2-Cys PRDXs (PRDX1–5) and 1-Cys PRDX (PRDX6), based on the number of cysteine residues on the active site (Rhee et al., 2001). PRDX6 is the only PRDX enzyme with both phospholipase A ${}_{2}$ (PLA ${}_{2}$ ) activity and glutathione peroxidase activity. The glutathione peroxidase activity of PRDX6 is vital in protecting sperm from ROS-induced oxidative damage and also plays a role in signal transduction to maintain sperm viability and fertilization (Chatterjee et al., 2011; O'Flaherty, 2018). Inhibition of the PLA ${}_{2}$ activity of PRDX6 in mice (Moawad et al., 2017) and humans (Fernandez and O'Flaherty, 2018) has a strong adverse effect on sperm viability and DNA oxidation. In rats, overexpression of PRDX1 and PRDX6 in the epididymal epithelium can protect sperm from oxidative stress (Wu et al., 2020). Moreover, knockout of PRDX6 results in abnormal sperm maturation, poor motility, and abnormal chromatin (Ozkosem et al., 2015). PRDX6 in the epididymis is therefore important for sperm maturation and protection; however, details about the role of PRDX6 in the epididymis remain elusive. In this study, we examined the expression and distribution of PRDX6 in epididymal tissue, epididymal fluid, and sperm of sheep.

## 2 Materials and methods

### 2.1 Materials

All chemicals and reagents were purchased from Sigma-Aldrich (St Louis, MO, USA) unless otherwise indicated.

### 2.2 Ethical statement

The protocols using animals in this study were approved by the Ethics Committee of Experimental Animals of Inner Mongolia Agricultural University, Inner Mongolia Autonomous Region, China (no. XMXK20180016).

### 2.3 Sample collection

Testicles coated with intact tunica albuginea from six healthy male small-tailed Han sheep 1 to 2 years of age were collected from a slaughterhouse (Hohhot) during the breeding season (September to November), stored in phosphate-buffered saline (PBS) containing 100 IU mL ${}^{-1}$ (where IU is International Unit) penicillin and 100 µ g mL ${}^{-1}$ streptomycin (15070063, Gibco, CA, USA), and brought to the laboratory within 2 h. Epididymal fluid and epididymal sperm were subsequently collected as described by Rana et al. (2017). The donor sheep were equally divided into three biological replicate groups, with two sheep (i.e., four testicles) within each group. The caput epididymis, corpus epididymis, and cauda epididymis were separated on an ultra-clean bench and immersed in 3 mL PBS on a sterile Petri dish (Corning Incorporated, NY, USA). Three to four incisions were made in each epididymal section with thin scissors to allow fluid in the ductus epididymis to leak out. Epididymal tissue was mechanically ground in liquid nitrogen, added to 1 mL lysis buffer (AR0105, Boster, Wuhan, China) with 1 % PMSF (phenylmethylsulfonyl fluoride) (G2008, Service-Bio, Wuhan, China), fully lysed for 30 min on ice, and centrifuged at 12 000 g for 5 min at 4 °C. The supernatant was then extracted and stored at -80 °C for subsequent use. In addition, luminal fluid containing sperm was collected from each part of the epididymis and centrifuged at 1000 g for 5 min at room temperature. The resulting supernatant was collected as epididymal fluid, and the sperm pellet was collected and washed three times with PBS. Sperm smears were stained using Leishman stain to ensure that sperm were not contaminated with blood cells (Garbyal et al., 2006).

### 2.4 Fresh semen collection

Semen was collected from three healthy sheep approximately 2 years of age with normal fertility using the fake vagina method. The semen was transferred to 1.5 mL centrifuge tubes, stored at 37 ° C, and brought back to the laboratory within 2 h for later use.

**Table 1:** Primers used for reverse transcription polymerase chain reaction (RT-PCR).

Gene	Sequence source	Primer sequence (5 ${}^{\prime }$ –3 ${}^{\prime }$ )	Annealing temperature	Product size
PRDX6	NM_001280704.1	F:CTTCACCAAAGAGCTCCCGT	58 ° C	107 bp
		R:TGGCTCATGGTGCTAAGTGG		
β -actin	NM_001009784.1	F:GTCATCACCATCGGCAATGA	58 ° C	88 bp
		R:CGTGAATGCCGCAGGATT		

Note: bp: base pair; PRDX6: Peroxiredoxin-6.

### 2.5 RNA extraction and reverse transcription quantitate PCR (RT qPCR)

Total RNA was extracted from different segments of epididymal tissue using TRIzol reagent (ET111-01-V2, TransGen, Beijing, China). Then, cDNA was synthesized using the PrimeScript™ RT Reagent Kit with gDNA Eraser (RR047A, TaKaRa, Shiga, Japan) according to the manufacturer's instructions.

The mRNA expression of PRDX6 in different segments of epididymal tissue was detected using SYBR Premix Ex Taq™ II (RR820A, TaKaRa) and primers that were designed and synthesized (Sangon, Shanghai, China) according to the sheep cDNA sequence published in GenBank (Table 1).

Using the β -actin steward gene as an internal reference, the 2 ${}^{-\Delta \Delta \mathrm{Ct}}$ method was used to calculate the mRNA expression levels of PRDX6 in different segments of the epididymis relative to the caput epididymis.

### 2.6 Total protein extraction and western blot analysis

Protein was extracted using CelLytic buffer with protease inhibitor cocktail (1860932, Thermo Scientific, Waltham, MA, USA) and phosphatase inhibitor cocktail (1862495, Thermo Scientific). After ultrasonic cracking, the samples were centrifuged at 14 000 g for 15 min, and the supernatant was collected. The protein concentration was determined using a bicinchoninic assay protein concentration determination kit (AR0146, Boster, Wuhan, China) according to the manufacturer's instructions. Absorbance values for sheep epididymal samples were detected at a wavelength of 562 nm. A standard curve was constructed, and protein concentrations were calculated for each sample.

Gels (12 % separated gels and 5 % concentrated gels) were prepared, and 30 µ g of protein samples was added for SDS-PAGE electrophoresis. The separated proteins were transferred to a polyvinylidene fluoride (PVDF) membrane by blotting. After sealing with 5 % skimmed milk powder sealing solution (SW3010, Solarbio, Beijing, China), anti-peroxiredoxin 6 antibody (ab73350, Abcam, Cambridge, MA, USA) was added to the membrane at 1:400 dilution, incubated for 12 h at 4 °C, and washed five times with 10× Tris-buffered saline containing Tween-20 (SL1328, Coolaber, Beijing, China). The horseradish peroxidase (HRP) sheep anti-rabbit immunoglobulin G (IgG) secondary antibody (BA1054, Boster) was then added at 1:800 dilution and incubated for 1 h at room temperature. Signals were revealed using a DAB color development kit (SK2020, Coolaber, Beijing, China), with incubation in the dark at room temperature for 5 min until the appearance of a darker color. Images were scanned using a gel-imaging device (Bio-Rad, Hercules, CA, USA), and expression of PRDX6 protein was analyzed in comparison to the β -actin steward gene as an internal reference.

### 2.7 Immunofluorescence of epididymal sperm and fresh sperm

Sperm were washed with PBS and fixed with 4 % paraformaldehyde. Smears were then made and permeabilized with 0.5 % Triton-100 for 15 min, incubated with 3 % H ${}_{2}$ O ${}_{2}$ for 10 min, blocked with 5 % BSA (bovine serum albumin) for 1 h, and incubated with primary antibody ( 1:100 , Abcam) overnight and secondary antibody ( 1:200 , Boster) for 1 h. Anti-fluorescent attenuation reagent containing 4 ${}^{\prime }$ ,6-diamidino-2-phenylindole was added, and photos were taken using a confocal microscope (Olympus, Tokyo, Japan).

### 2.8 Statistical analysis

Data are shown as the mean ± standard error, and three biological replicates were required for all experiments. PRDX6 mRNA and protein expression data from different groups were analyzed by one-way analysis of variance in SPSS software (SPSS 25.0 Inc., Chicago, IL, USA). 

## 3 Results

### 3.1 Expression of PRDX6 mRNA and protein in different segments of epididymal tissue

**Figure 1:**
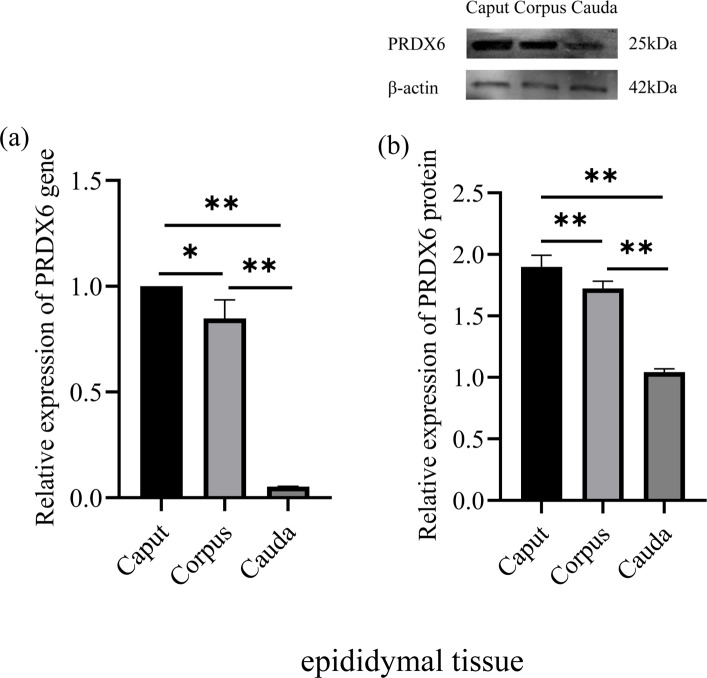
The expression levels of PRDX6 mRNA **(a)** and protein **(b)** in epididymal tissue from the caput, corpus, and cauda epididymis. The expression values for qPCR and western blot are given relative to the expression level of PRDX6 in the caput epididymis and the expression level of β -actin, respectively. The bars represent the mean ± standard error from three independent biological replicates. ${}^{*}p<0.05$ ; ${}^{**}p<0.01$ .

The expression of *PRDX6* mRNA was higher in the caput epididymal tissue ( 1.02±0.19 ) than in the corpus epididymal tissue ( 0.82±0.07 , p<0.05 ) and the cauda epididymal tissue ( 0.05±0.00 , p<0.01 ; Fig. 1a). Likewise, the expression of PRDX6 protein was also higher in the caput epididymal tissue ( 1.90±0.08 ) than in the corpus epididymal tissue ( 1.72±0.05 , p<0.01 ) and the cauda epididymal tissue ( 1.04±0.02 , p<0.01 ; Fig. 1b). Thus, the epididymis expression of *PRDX6* mRNA and protein showed a decreasing trend from the caput epididymis to the cauda epididymis.

### 3.2 Levels of PRDX6 protein in different regions of the epididymal fluid

**Figure 2:**
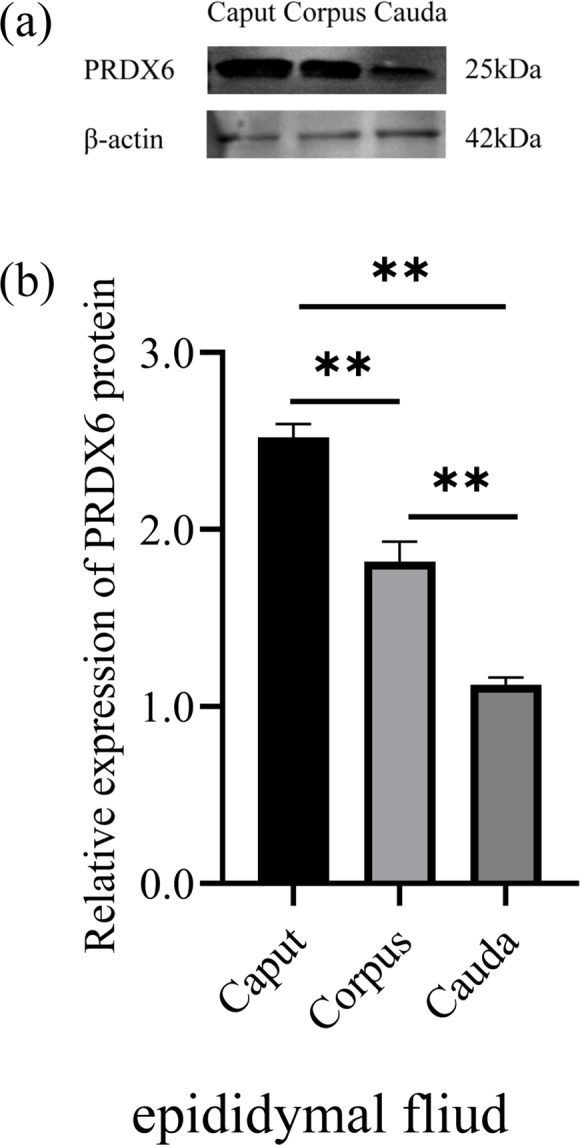
The expression levels of PRDX6 protein in epididymal fluid from the caput, corpus, and cauda epididymis. **(a)** Western blot analysis of PRDX6. **(b)** Relative protein level of PRDX6. The bars represent the mean ± standard error from three independent biological replicates. ${}^{*}p<0.05$ ; ${}^{**}p<0.01$ .

The level of PRDX6 protein in epididymal fluid from the caput epididymis ( 2.52±0.06 ) was higher than that in fluid from the corpus epididymis ( 1.82±0.09 , p<0.01 ) and the cauda epididymis ( 1.12±0.03 , p<0.01 ; Fig. 2). Thus, the level of PRDX6 protein in epididymal fluid showed a decreasing trend from the caput epididymis to the cauda epididymis.

### 3.3 Expression of PRDX6 in sperm from different parts of the epididymis

**Figure 3:**
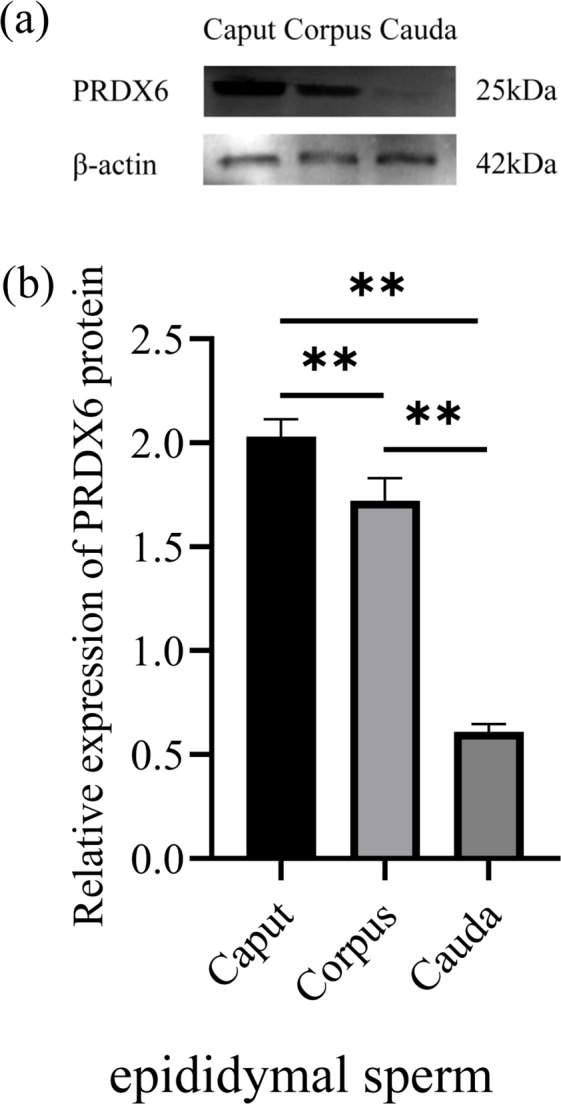
The expression levels of PRDX6 protein in epididymal sperm from the caput, corpus, and cauda epididymis. **(a)** Western blot analysis of PRDX6. **(b)** Relative protein level of PRDX6. The bars represent the mean ± standard error from three independent biological replicates. ${}^{*}p<0.05$ ; ${}^{**}p<0.01$ .

The expression of PRDX6 protein in sperm from the caput epididymis ( 2.03±0.07 ) was higher than that in sperm from the corpus epididymis ( 1.72±0.09 , p<0.01 ) and cauda epididymis ( 0.11±0.03 , p<0.01 ; Fig. 3).

### 3.4 Distribution of PRDX6 in sperm cells in the epididymis and semen

**Figure 4:**
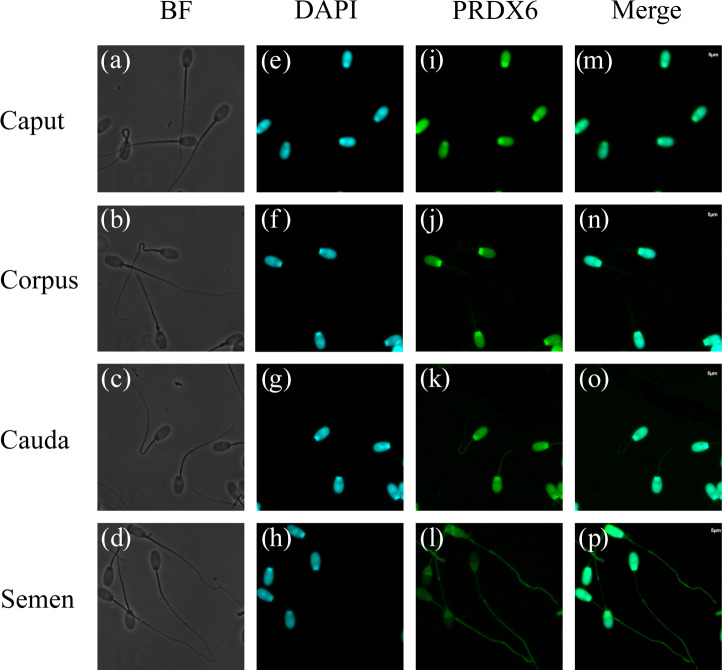
Immunofluorescence analysis of PRDX6 in sperm from the caput, corpus, and cauda epididymis and semen. **(a–d)** bright-field microscopy, **(e–h)** DAPI (4 ${}^{\prime }$ ,6-diamidino-2-phenylindole) staining, **(i–l)** PRDX6 staining, **(m–p)** merged images. Scale bars are 5 µ m.

Immunofluorescence results for PRDX6 in sperm are shown in Fig. 4. PRDX6 protein was strongly expressed only in the head of sperm from the caput epididymis and corpus epididymis. By contrast, after the sperm was transported into the cauda epididymis, PRDX6 protein could be detected in the head, middle, and principal pieces of the tail. In ejaculated sperm, PRDX6 was distributed in all parts of the cell.

## 4 Discussion

The luminal microenvironment of the epididymis is created by factors that are synthesized and secreted by epithelial cells (Chen et al., 2021). This microenvironment participates in sperm plasma-membrane modification and provides a place for sperm maturation, storage, and protection. As sperm pass through the epididymis, they interact with proteins in the epididymal fluid that contribute to maturation (Guyonnet et al., 2011; Barrachina et al., 2022). During sperm maturation, a dynamic balance is maintained between various antioxidant enzymes and ROS in the ductus epididymis. If this balance is disturbed, the sperm undergo serious oxidative damage (Mahfouz et al., 2010). All mammalian sperm need a set of antioxidant enzymes to control ROS during maturation; however, the expression of different antioxidant enzymes varies among species. SOD activity was demonstrated to rise gradually from the caput epididymis to the cauda epididymis in the epididymal sperm of boars (Park et al., 2012) and the epididymal fluids of miniature breed stallions (Bustamante-Filho et al., 2014). Conversely, GPX5 expression decreased rapidly from the caput epididymis to the cauda epididymis in small-tailed Han sheep (Li et al., 2018). The activity of CAT was reported to increase progressively from the caput epididymis to the cauda epididymis in miniature breed stallions (Bustamante-Filho et al., 2014), whereas it was lowest in the cauda epididymis compared with other regions in European bison (Koziorowska-Gilun et al., 2013). The antioxidant pathways of each enzyme are different, and the varied expression of different enzymes may reflect a complex synergistic process to protect sperm in the epididymis (O'Flaherty, 2019).

PRDXs can be involved in the antioxidant protection and help regulate redox homeostasis (Wood et al., 2003a, b). They are highly sensitive to oxidative stress and become oxidized by ROS in a dose-dependent manner (O'Flaherty and de Souza, 2011). As a member of the PRDX family, PRDX6 has a greater potency in preventing lipid peroxidation compared with other antioxidant enzymes due to its PLA ${}_{2}$ activity and peroxidase activity. Knockout of the PRDX6 gene revealed that male mice lacking PRDX6 were less fertile than wild-type mice (Ozkosem et al., 2015). This loss of fertility is associated with impaired sperm motility, DNA oxidation, reduced sperm protein levels, and DNA compression (O'Flaherty, 2018; Fernandez and O'Flaherty, 2018).

As sperm transit through the epididymis, they encounter different epididymal microenvironments that promote sperm maturation (Dacheux and Dacheux, 2013). Although expression patterns of various antioxidant enzymes in the epididymis of different animals have been reported, there are no reports on PRDX6 expression in the epididymis. In this study, we found that expression of PRDX6 in the caput epididymis was higher than that in the corpus epididymis and cauda epididymis, and this was accompanied by a downward trend in the PRDX6 protein content of the epididymal fluid from the caput epididymis to the cauda epididymis. This may be due to high levels of ROS in the cauda epididymis. Scavenging of ROS in sperm by PRDX6 requires interconversion between the oxidation and reduction states of PRDX6, but excessively high ROS levels cause the majority of PRDX6 to become peroxidized and form inactive, high-molecular-mass complexes, resulting in loss of PRDX6 antioxidant function (O'Flaherty, 2019). Liu and O'Flaherty (2017) also found that PRDX6 expression was higher in the caput epididymis than in the cauda epididymis in rats. These results indicate that different parts of the epididymis have different demands for PRDX6, and regional differential expression of antioxidant enzymes in the epididymis is crucial for the regulation of redox potential during sperm maturation and storage in the epididymal microenvironment (Žaja et al., 2016).

Mammalian sperm undergo some structural modifications during maturation, which are important for biological processes such as metabolism, capacitation, and fertilization (Gervasi and Visconti, 2017). We found that PRDX6 was present only in the head of sperm in the caput epididymis and corpus epididymis but began to be distributed also in the principal and middle regions of the tail after transport into the cauda epididymis. Moreover, PRDX6 was distributed in all parts of sperm after ejaculation. The presence of PRDX6 in the tail and flagella-enriched fractions of human sperm might play a role in protecting vital components of these complex structures against oxidative stress (O'Flaherty and de Souza, 2011). Although the expression level of PRDX6 decreases from the caput epididymis to the cauda epididymis in sheep, its wide intracellular distribution in sperm cells in the cauda epididymis suggests that it plays an important role in providing antioxidant protection.

## 5 Conclusions

PRDX6 is expressed in the epididymal tissue, epididymal fluid, and epididymal sperm of sheep. As the sperm mature and transit through the epididymis, the intracellular distribution of PRDX6 gradually becomes more extensive. This suggests that PRDX6 has an important role in promoting sperm maturation. Further research is needed to determine the detailed mechanism of action of PRDX6 during sperm maturation.

## Data Availability

The data are available from the corresponding author upon request.
